# The Role of Nrf2: Adipocyte Differentiation, Obesity, and Insulin Resistance

**DOI:** 10.1155/2013/184598

**Published:** 2013-09-08

**Authors:** Hyun-Ae Seo, In-Kyu Lee

**Affiliations:** ^1^Department of Internal Medicine, Daegu Fatima Hospital, 99 Ayang-ro, Dong-gu, Daegu 701-600, Republic of Korea; ^2^Departments of Internal Medicine and Biochemistry and Cell Biology, Research Institute of Aging and Metabolism, WCU Program, Kyungpook National University School of Medicine, 50 Samduk-2Ga, Jung-gu, Daegu 700-721, Republic of Korea

## Abstract

Metabolic diseases, such as type 2 diabetes and obesity, are increasing globally, and much work has been performed to elucidate the regulatory mechanisms of these diseases. Nuclear factor E2-related factor 2 (Nrf2) is a basic leucine zipper transcription factor that serves as a primary cellular defense against the cytotoxic effects of oxidative stress. Recent studies have proposed a close relationship between oxidative stress and energy metabolism-associated disease. The Nrf2 pathway, as a master regulator of cellular defense against oxidative stress, has emerged as a critical target of energy metabolism; however, its effects are controversial. This review examines the current state of research on the role of Nrf2 on energy metabolism, specifically with respect to its participation in adipocyte differentiation, obesity, and insulin resistance, and discusses the possibility of using Nrf2 as a therapeutic target in the clinic.

## 1. Introduction 

Nrf2, a basic leucine zipper transcription factor, is encoded by the NFE2L2 gene in humans [1]. Under normal conditions, Nrf2 is sequestered in the cytoplasm by associating with kelch-like ECH-associated protein 1 (Keap1) and cullin 3. Cullin 3 ubiquitinates Nrf2. Keap1 is a substrate of cullin 3, which facilitates the ubiquitination of cullin 3 [2]. During oxidative stress, Keap1 senses cellular oxidative stress and releases Nrf2, at which time Nrf2 travels to the nucleus. In the nucleus, Nrf2 forms a complex with Maf and Jun proteins and binds to the antioxidant response element (ARE) in the upstream promoter region found in many antioxidative genes which initiate their transcription [3]. In this way, Nrf2 is working as a primary cellular defender against the cytotoxic effects of oxidative stress.

Oxidative stress results in the development of many diseases, including neurodegenerative diseases, tumors, and metabolic syndromes [4]. Recent studies have shown that oxidative stress is closely associated with energy metabolism [5–9]. When obese mice are treated with an NADPH oxidase inhibitor, reactive oxygen species (ROS) production in adipose tissue is reduced, adipocytokine dysregulation is attenuated, and diabetes, hyperlipidemia, and hepatic steatosis are improved [5]. Systemic oxidative stress leads to a lower aerobic capacity and impaired skeletal muscle energy metabolism in patients with metabolic syndrome [6]. Obese people exhibit an increase in systemic oxidative stress [8]. When obese people were subjected to restricted energy intake, oxidative stress marker levels were lower and antioxidant levels were higher than when they were not subjected to energy intake restriction. Subsequently, decreased oxidative stress was found to decrease metabolic syndrome by about 50% [9]. 

The Nrf2 pathway, which counteracts oxidative stress, also plays an important role in energy metabolism; however, its effects on energy metabolism are controversial. Here, we discuss the current state of research on the role of Nrf2 in energy metabolism, specifically with respect to its participation in adipocyte differentiation, obesity, and insulin resistance. In addition, we suggest that an Nrf2 agonist would be a promising target for the treatment of metabolic syndromes.

## 2. Nrf2-Adipocyte Differentiation

In the past, adipocytes were thought to function only in fat storage. Today, adipose tissue is defined as an important endocrine organ [10]. Understanding the regulatory mechanisms of adipocyte differentiation is the basis for research on metabolic diseases and the pharmaceutical intervention. Adipocyte differentiation is a very complicated process that involves a network of transcription factors and adipocyte marker genes [11, 12]. Increasing evidence shows that oxidative stress is an important factor in adipogenesis [13, 14]. To elucidate the function of Nrf2 in adipogenesis, many investigators have generated Nrf2 or Keap1 knockout (KO) mice and manipulated 3T3-L1 cells with Nrf2 activators; however, results are mostly inconsistent, and there is no consensus on the role of Nrf2 in adipocyte differentiation. 

Pi et al. found that wild-type (WT) mice had marked enlargement of fat pads and increased white adipose tissue weight compared with Nrf2 KO mice, after 12 weeks on the high fat diet (HFD). To identify the relationship between Nrf2 and adipogenesis, the authors compare the adipocyte differentiation capacity of mouse fibroblasts isolated from wild-type embryos with those from Nrf2-deficient embryos. In the absence of Nrf2, adipocyte differentiation was suppressed, resulting from the downregulation of PPAR*γ* and CEBP*α* expression. They also performed shRNA-mediated knockdown (KD) of endogenous Nrf2 in 3T3-L1 cells and human subcutaneous preadipocytes. Adipogenesis in these cells was suppressed by KD of Nrf2. And KD of Keap1 in 3T3-L1 cells results in activation of Nrf2 and an enhanced adipogenesis [15]. In a subsequent study, Hou et al. show Nrf2 regulation of C/EBP*β* in preadipocytes derived from white adipose tissue of Nrf2 KO and wild-type mice [16]. In the experiments using 3T3-L1 cells with Nrf2-KD, Keap1-KD, or Double-KD, adipocyte differentiation was impaired in accordance with the lack of Nrf2 expression [16]. Their finding consistently illustrates Nrf2 augmenting adipocyte differentiation [15, 16]. 

Conflicting results have also been reported. The AHR pathway is associated with the impaired differentiation of 3T3-L1 preadipocytes and mouse embryonic fibroblasts (MEFs) to mature adipocytes [17, 18]. Shin et al. suggest that Nrf2 pathway inhibits adipogenesis via activation of the aryl hydrocarbon receptor (AHR) pathway [19]. RT-PCR and immunoblotting assays show higher AHR mRNA and protein levels in Nrf2+/+ MEFs than in Nrf2−/− MEFs. To identify the effects of Nrf2 on adipocyte differentiation, the authors monitored intracellular lipid accumulation microscopically. Lipid droplets were clearly detectable in Nrf2−/− MEFs with elevated expression of CEBP*α* and FABP4 at 3 days after stimulation, whereas droplets were first visible 5 days after stimulation of Nrf2+/+ MEFs. Adipocyte differentiation was also delayed in Keap1−/− MEFs compared with Keap1+/+ MEFs [19]. In addition, Xu and colleagues demonstrate that Nrf2 activation in Keap1-KD and OBKeap1-KD MEFs suppressed adipogenesis and decreased adipogenic genes, such as PPAR*γ*, CEBP*α*, and FABP4. MEFs from WT and OB mice were pretreated with sulforaphane to identify the effect of pharmacological Nrf2 activation in adipocyte differentiation. Pharmacological Nrf2 activation by sulforaphane prevented adipogenesis and lipid accumulation [20]. Some substances that have the ability to stimulate Nrf2 have been reported to inhibit adipogenesis. Carnosic acid (CA) and carnosol (CS) originating from dried rosemary leaves activate Nrf2 pathway [21]. These two potent compounds decrease the number of cells containing lipid droplets in 3T3-L1 cells dose-dependently through Nrf2 pathway in the study of Takahashi et al. [22]. The authors performed a microarray analysis in order to detect genes induced by CA. The majority of the genes increased by CA were phase II enzymes which are involved in the metabolism of GSH. They suggested that induction of phase II enzymes and stimulation of GSH metabolism are one of the mechanisms of the inhibition of adipocyte differentiation by CA and CS [22].

In another study, Shin et al. show Nrf2 to have no effects on adipocyte differentiation. They investigate CEBP*α* and FABP4 mRNA levels in white adipose tissue (WAT) of Nrf2-disrupted mice and wild-type mice fed a control or HFD for 13 weeks. There was no difference in the expression of these adipogenic markers between wild-type and Nrf2-disrupted mice. Moreover, CEBP*α* and FABP4 expression was not affected by an Nrf2 activator, the synthetic oleanolic triterpenoid 1-[2-cyano-3,12-dioxooleana-1,9(11)-dien-28-oyl]imidazole (CDDO-Im) [23]. As this result differs from their previous *in vitro* experiment [19], they suggested that a prolonged feeding period is required to detect the effects of the Nrf2 genotype on adipogenesis in animal models. 

Suggested mechanisms of regulation of Nrf2 in adipocyte differentiation are adipogenic gene regulation, the regulation of AHR pathway, and stimulation of GSH metabolism. Experiments addressing the role of Nrf2 in adipogenesis did not yield similar results (Table 1). These discrepancies may be due to differences in cell lines, cell passage number, animals used, diet composition, feeding period, and so on. Nonetheless, it is clear that Nrf2 plays critical roles in adipose tissue. Adipose tissue secretes a variety of bioactive peptides known as adipokines, such as adiponectin and resistin, and expresses numerous receptors [24]. Through these actions, adipose tissue is involved in various biological processes, including energy metabolism, and neuroendocrinological and immunological functions [24]; therefore, adipocytes are beneficial, and an appropriate amount of fat tissue is critical for normal homeostasis in mammals. Adverse metabolic consequences result when adipose tissue is excessive or scarce [25, 26]. Therefore, it is important to investigate the relationship between Nrf2 and adipocyte differentiation so that the systemic changes it elicits on metabolism can be addressed in parallel.

## 3. Nrf2 Obesity

Obesity can result in obesity-related diseases, such as type 2 diabetes, osteoarthritis, and cardiovascular diseases. These diseases have a significant negative impact on health, mortality, and the cost of running public health systems. Currently, there are many efforts to reduce obesity. Oxidative stress is one of the key factors involved in obesity-related morbidity, and Nrf2 may be a promising drug target to treat obesity [5]. However, many published studies using Nrf2 KO and Keap1-KD mice, as well as an Nrf2 agonist, show inconsistent results on the role of Nrf2 in obesity.

The results of research into the effects of Nrf2 on obesity can be classified into two groups according to the method used to regulate Nrf2. One approach uses an Nrf2 activator, which stimulates Nrf2 signaling intermittently, and the other uses Nrf2 KO or Keap1-KD mice, which leads to permanent up- or downregulation of Nrf2.

The effects of Nrf2 activator on obesity were first reported by Shin et al. [23]. These authors demonstrate that the CDDO-Im, a potent Nrf2 activator, prevented body weight gain. Wild-type mice (C57BL/6J) fed a control or HFD were dosed with vehicle or 30 *μ*mol/kg CDDO-Im. Body weight and the amount of WATs were smaller in mice fed with CDDO-Im compared to mice fed without CDDO-Im in HFD condition [23]. To determine whether this inhibitory effect of CDDO-Im was Nrf2-dependent, the same experiment was repeated using Nrf2 disrupted mice. The inhibitory effect of CDDO-Im on weight gain was completely lost in Nrf2 disrupted mice fed an HFD; however, the rate of body weight increment following an HFD and weights of fat tissue were smaller in Nrf2 disrupted mice than in WT mice regardless of CDDO-Im. [23]; thus, the authors hypothesized that Nrf2 activation by CDDO-Im negatively affects fat accumulation, and constitutive levels of Nrf2 may contribute to accumulation of fat following an HFD. Subsequently, several natural and synthetic substances with the ability to stimulate Nrf2 signaling have been reported to be effective against obesity. The report by Yu et al. investigated the effect of oltipraz and suggested that the Nrf2 activator prevents insulin resistance and obesity caused by an HFD [27]. C57BL/6J mice were fed with a low fat diet, HFD, or HFD plus oltipraz for 28 weeks. Oltipraz administration rescued the effect of HFD on body weight gain during the entire experimental period. The decrease in Nrf2 expression level in mice fed an HFD was partially restored by oltipraz [27]. Additionally, some substances, such as ellagic acid, quercetin, curcumin, resveratrol, and chromium histidinate, have antiobesity effects. Although each of these compounds has unique characteristics, it was suggested that Nrf2 activation was one of the underlying mechanisms for the effects of these substances on obesity [28–33]; thus, the activation of Nrf2 by an Nrf2 agonist could constitute the basis of a therapy for obesity.

Experiments using Nrf2 KO or Keap1-KD mice, which have a constitutive altered Nrf2 pathway, showed various effects on obesity. Pi and colleagues challenged Nrf2 KO and wild-type mice with an HFD. After 12 weeks, Nrf2 KO mice gained 15% of their starting weight, whereas wild-type mice gained 75% of their starting weight. Differences in food consumption, intestinal fat absorption, and physical activity were not observed; instead, an increase in fat pad content was noted in wild-type mice. This result suggests that Nrf2 deficiency protects against diet-induced obesity [15]. In the study by Chartoumpekis et al., wild-type and Nrf2 KO mice were fed an HFD for 180 days, and both groups gained body weight initially at about the same rate; however, after 90 days, wild-type mice gained more weight than Nrf2 KO mice. The authors concluded that Nrf2 deficiency protects against HFD-induced obesity [34]. They suggested fibroblast growth factor 21 (FGF21) to protect Nrf2 KO mice from obesity [34]. The opposite result was also reported. Keap1-KD mice have increased Nrf2 activity. When wild-type and Keap1-KD mice were fed a standard or HFD for 36 days, Keap1-KD mice had lower body weights and epididymal fat masses than wild-type mice. This study showed a constitutive increase in Nrf2 to positively affect HFD-induced weight gain [20]. Conversely, two reports have shown no effect of Nrf2 on obesity [35, 36]. Zhang et al. found that feeding an HFD to Nrf2-null, wild-type, and Keap1-KD mice for 12 weeks led to no significant difference in body weight between the three groups [35]. In the experiment of Collins and colleagues [36], 2-month-old, male, low-density lipoprotein receptor-deficient mice transplanted with either wild-type or Nrf2-deficient bone marrow cells were fed a high fat and high cholesterol diet for 7 months. No significant change in weight or body fat percentage was observed between the two groups [36]. The study was designed to determine the role of hematopoietic Nrf2 in atherosclerosis and nonalcoholic steatohepatitis. The low-density lipoprotein receptor-deficient mice developed both advanced atherosclerosis and all of the components of nonalcoholic steatohepatitis when they were fed an HFD. We suggested that differences in the experimental design, such as the fat sources of the diet, the duration of HFD feeding, gender, and the genetic background of the mice, were responsible for the discrepancies on the role of Nrf2 in obesity. 

Except for two contradictory reports [35, 36], Nrf2 deficiency in Nrf2 KO mice leads to weight loss [15, 23, 34], and an increase in Nrf2 activity in Keap1-KD mice also leads to weight loss [20]. The main cause of difference between Nrf2 KO and Keap1 KD may be due to the method used to alter the regulation of Nrf2. Keap1 KD elevated Nrf2 activity, but it could have other effects that are independent of its effects on Nrf2 activity. The conflicting results may also be the result of differences in diet composition, feeding duration, or mouse strains. 

In summary, intermittent Nrf2 activation reduces total body weight and fat tissue content under HFD conditions, whereas KO or KD of Nrf2 leads to various, often conflicting outcomes (Table 2). And the underlying mechanism of Nrf2 in obesity is not clear. Although some studies suggested FGF21 as metabolic regulator, most of the study presented only the change of body weight and fat mass without mechanism. Therefore, future studies are needed to elucidate the mechanism of Nrf2 in obesity.

## 4. Nrf2-Insulin Resistance

Increased oxidative stress, chronic inflammation, and insulin resistance are manifestations of several chronic diseases, including obesity, type 2 diabetes, and metabolic syndromes [4]. Recent studies showed a close relationship between these conditions and Nrf2 modulation. Nrf2 upregulated the expression of Heme oxygenase 1 (HO-1), glutathione peroxidase, glutathione *S*-transferase A1, NAD(P)H, quinone oxidoreductase, and glutamate-cysteine ligase. All of these proteins maintain redox homeostasis and cell viability in response to oxidative stress [37, 38]. On the other hand, glycogen synthase kinase 3 beta (GSK-3*β*) attenuated both phase II gene expression and oxidant protection. GSK-3*β*, which is inhibited by insulin-Akt-mediated phosphorylation, phosphorylates Nrf2 through Fyn phosphorylation and exports Nrf2 from the nucleus [39]. Oxidative stress is a main inducer of insulin resistance [40]; thus, although the precise mechanisms are not completely understood, Nrf2 is associated with insulin signaling pathway and insulin resistance. 

As in obesity, studies about the effect of Nrf2 on insulin resistance can also be classified into two groups according to the way Nrf2 regulation was studied. One method employed a substance that stimulates Nrf2 signaling intermittently, and the other used Nrf2 KO or Keap1-KD mice, which have a constant absence or high levels of Nrf2. 

Most studies using Nrf2 KO or Keap1-KD mice have shown that Nrf2 deficiency has a positive effect on glucose homeostasis. Chartoumpekis et al., as previously mentioned, fed wild-type and Nrf2 KO mice with an HFD for 180 days [34]. Nrf2 KO mice were more glucose-tolerant and insulin-sensitive than wild-type mice through the regulation of FGF21 expression [34]. Lep(ob/ob)-Keap1-KD mice showed higher glucose concentration than Lep(ob/ob) mice by glucose and insulin tolerance tests, and Nrf2 regulates genes involved in glucose uptake and tolerance [20]. These results implicate that Nrf2 activation may impair insulin signaling and glucose uptake. In the study of Zhang et al., an intraperitoneal glucose tolerance test (GTT) was used to evaluate insulin resistance. Nrf2-null mice maintained low blood glucose concentrations and normal glucose clearance when an HFD was fed. Thus, the deficiency of Nrf2 seems to protect mice from HFD-induced hyperglycemia [35]. There is one paper to report the importance of Nrf2 in nonmyeloid cells. Global Nrf2 KO and myeloid-selective Nrf2 deficient mice were fed a standard or HFD for 10 weeks [41]. Insulin tolerance tests showed global Nrf2 KO mice to be more sensitive to insulin than wild-type mice, irrespective of diet. On the other hand, myeloid-selective Nrf2 deficiency mice were less sensitive to insulin. The authors suggested that nonmyeloid Nrf2 is essential and acts negatively for the development of insulin resistance via the regulation of inflammatory gene expression [41]. Aleksunes et al. reported conflicting results. In their study, male wild-type and Nrf2-null mice received a single intraperitoneal injection of streptozotocin (200 mg/kg) to induce type 1 diabetes. Compared with wild-type mice, mice lacking Nrf2 have lower basal serum insulin levels and prolonged hyperglycemia in response to an intraperitoneal glucose challenge. They suggested that Nrf2 deletion impairs glucose tolerance and exacerbates hyperglycemia in type 1 diabetic mice [42]. The reason for the discrepancy with previous studies showing the negative effect of Nrf2 in insulin resistance may be the type of diabetes studied; Aleksunes group induced type 1 diabetes with streptozotocin, but most of the aforementioned studies induced insulin resistance by feeding mice an HFD like type 2 diabetes [34, 42]. The pathogenesis of type 1 and type 2 diabetes is different. Type 1 diabetes results from beta-cell destruction, whereas insulin resistance and abdominal insulin secretion are central to the development of type 2 diabetes [43]; therefore, the effects of oxidative stress on insulin resistance are likely to be different based on the type of diabetes. The effect of the Nrf2 stimulation by environmental oxidative stress in insulin resistance was reported [44]. Exposure to environmental inorganic arsenic (iAs) is known to cause oxidative stress [45]. iAs exposure suppressed insulin-stimulated phosphorylation of AKT at serine residue 473, glucose uptake, and glucose transporter type 4 (GLUT4) expression in 3T3-L1 adipocytes in keeping with the increased Nrf2 expression. These results suggest that iAs-induced insulin resistance in adipocytes results from Nrf2 activation and a subsequent induction of antioxidant enzymes [44]. Nrf2 KO mice, Keap1-KD mice, and mice exposed to chronic stress are common in that they stimulate Nrf2 constantly. Most of these studies, except for one [42], have shown that Nrf2 acts negatively on insulin resistance. 

The Nrf2 agonist transiently activates Nrf2. Weisberg et al. studied the effects of curcumin on diabetes. Wild-type and *ob/ob* male mice were fed a standard or HFD with or without curcumin. The HFD-induced obese mice and leptin deficient *ob/ob* mice with curcumin showed improved glucose metabolism as determined by random glucose level, glucose tolerance testing, insulin tolerance testing, and hemoglobin A1c percentage [31]. While there was no discussion on Nrf2 expression in this paper, curcumin can activate Nrf2 [46]. In addition, He et al. recently showed that curcumin improved insulin sensitivity through the regulation of muscular mitochondrial redox balance by Nrf2 signaling activation [30]. Several substances, which stimulate Nrf2 pathway and improve insulin resistance, were introduced. Oltipraz, which can activate the Nrf2 signaling pathway, improved glucose metabolism as demonstrated by intraperitoneal glucose tolerance test, intraperitoneal insulin tolerance test, and intraperitoneal pyruvate tolerance tests in C57BL/6J mice fed an HFD and prevented HFD-induced impairments in insulin signaling and GLUT4 depletion in adipose tissue. Oltipraz restored decreased AMP-activated protein kinase (AMPK) signalling, increased p70S6 kinase, and decreased GLUT4 expression [27]. Ellagic acid also increased the levels of Nrf2 and attenuated a high carbohydrate, HFD-induced metabolic syndrome in rats. An ellagic acid supplement lowered fasting blood glucose concentrations during an oral glucose tolerance test in HFD conditions [28]. Resveratrol is known to have a protective function against insulin resistance [33, 47]. Nrf2 agonist, Dh404, was reported to attenuate insulin resistance *in vitro* and *in vivo* cardiomyocytes through the regulation of extracellular signal regulated kinase (ERK) pathway [48]. Thus, transient increases in Nrf2 expression by Nrf2 agonist improve glucose homeostasis and insulin sensitivity. 

Reports on the role of Nrf2 in insulin resistance showed two conflicting results, and the suggested underlying mechanisms are diverse (Table 3). Nrf2 in Nrf2 KO, Keap1-KD mice and chronic oxidative stress negatively affect insulin resistance. The positive effect of Nrf2 in insulin resistance was demonstrated by activating Nrf2 intermittently with several substances. Such substances could be used as part of a therapy to prevent insulin resistance. 

## 5. Conclusions 

In this review, we discussed the current state of knowledge on the role of Nrf2 in adipocyte differentiation, obesity, and insulin resistance. Nrf2 activation in adipocytes results in diverse cellular events. Regarding obesity and insulin resistance, Nrf2 leads to similar results. An Nrf2 agonist improved obesity and insulin resistance. On the other hand, KD or KO of Nrf2 did not improve obesity or insulin resistance. The main cause of this difference appears to be the exhaustion of Nrf2 function when it is constantly activated. Thus, permanent up- or downregulation of Nrf2 may lead to Nrf2 pathway dysfunction and unfavorable outcomes. Some materials such as curcumin, oltipraz, and ellagic acid stimulated Nrf2 signaling transiently because this compound was fed as one component of meal or drug. Intermittent Nrf2 stimulation seems to defend cells against the oxidative stress effectively. 

In this review, the role of transcription factor proven in structural change, such as KO animal, is not equal to the function of it as agonist. Mammals, including human, strive continually to maintain homeostasis. When transcription factors or genes lose their functions, other factors will compensate. When hyperactivity is induced through constitutive or intermittent changes, cellular responses are variable. Thus, we propose that the role of Nrf2, found using KO mice, is different from its role as a drug target. 

Finally, Nrf2 plays a critical role in adipose tissue, and the effect of Nrf2 on adipocyte differentiation is needed to interpret in accordance with systemic metabolic changes. Also, we suggest that an Nrf2 agonist could be used to prevent obesity, and insulin resistance. Further studies to elucidate the underlying mechanism and to investigate adipocyte differentiation, obesity, and insulin resistance at the same time are required. 

## Figures and Tables

**Table 1:** The role of Nrf2 in adipocyte differentiation.

Adipogenesis and Nrf2	Suggested mechanism	Presented marker	Reference
Nrf2↓→adipogenesis↓	Regulation of adipogenic genes and adipogenesis downstream genes	PPAR*γ*, CEBP*α*, aP2, CD36, LPL, and ADPSN	[15]
Nrf2↓→adipogenesis↓	Regulation of adipogenic genes	PPAR*γ*1, PPAR*γ*2, CEBP*β*, Adipsin, and FABP4	[16]
Nrf2↑→adipogenesis↓	Regulation of adipogenic genes, Ahr and Ahr downstream genes	PPAR*γ*2, CEBP*α*, CEBP*β*, FABP4, Cyp1a1, Cyp1b1, and Gsta1	[19]
Nrf2↑→adipogenesis↓	Regulation of adipogenic genes, adipogenesis downstream genes, Ahr and Ahr downstream genes	PPAR*γ*, CEBP*α*, CEBP*β*, FABP4, FAS, SREBP1c, SCD-1, ACC-1, ACC-2. CD36, Cyp1b1, and Gsta1	[20]
Nrf2↑→adipogenesis↓	Induction of phase II enzymes and stimulation of GSH metabolism	Gsta2, Gclc, Abcc4, Abcc1, and GSH level	[22]
Nrf2↑→adipogenesis↔	Regulation of adipogenic genes	CEBP*α* and FABP4	[23]

**Table 2:** The role of Nrf2 in obesity.

Obesity and Nrf2	Regulation of Nrf2	Reference
Nrf2↓→obesity↓	Nrf2 KO mice	[15]
Nrf2↑→obesity↓	Keap1-KD, Lep(ob/ob)-Keap1-KD mice	[20]
Nrf2↑→obesity↓ Nrf2↓→obesity↓	Nrf2 agonist: CDDO-Im Nrf2 disrupted mice	[23]
Nrf2↓→obesity↓	Nrf2 KO mice	[34]
Nrf2↓→obesity↔	Nrf2-null mice, Keap1-KD mice	[35]
Nrf2↓→obesity↔	Myeloid Nrf2 KO mice	[36]
Nrf2↑→obesity↓	Nrf2 agonist: oltipraz	[27]
Nrf2↑→obesity↓	Nrf2 agonist: ellagic acid	[28]
Nrf2↑→obesity↓	Nrf2 agonist: quercetin	[29]
Nrf2↑→obesity↓	Nrf2 agonist: curcumin	[30, 31]
Nrf2↑→obesity↓	Nrf2 agonist: chromium histidinate	[32]
Nrf2↑→obesity↓	Nrf2 agonist: resveratrol	[33]

**Table 3:** The role of Nrf2 in insulin resistance.

Insulin resistance and Nrf2	Regulation of Nrf2	Suggested mechanism	Reference
Nrf2↑→insulin resistance↑	Keap1-KD, Lep(ob/ob)-Keap1-KD mice	Regulation of genes involved in glucose uptake and tolerance	[20]
Nrf2↓→insulin resistance↓	Nrf2-KO mice	FGF21	[34]
Nrf2↓→insulin resistance↓	Nrf2-null mice, Keap1-KD mice	Hepatic expression of insulin like growth factor binding protein-1 (Igfbp-1) and FGF21	[35]
Nrf2↓→insulin resistance↓	Global Nrf2-KO mice Myeloid-selective Nrf2 deficiency mice	Inflammatory gene expression	[41]
Nrf2↓→insulin resistance↑	STZ-treated Nrf2-null mice	Gluconeogenesis and Glycolysis-related genes	[42]
Nrf2↑→insulin resistance↑	Chronic iAs exposure	Inflammatory gene and GLUT4 expression	[44]
Nrf2↑→insulin resistance↓	Nrf2 agonist: oltipraz	AMPK signalling, p70S6 kinase, and GLUT4 expression	[27]
Nrf2↑→insulin resistance↓	Nrf2 agonist: ellagic acid	Attenuation of oxidative stress and inflammation in liver	[28]
Nrf2↑→insulin resistance↓	Nrf2 agonist: curcumin	Muscular mitochondrial redox balance	[30]
Nrf2↑→insulin resistance↓	Nrf2 agonist: curcumin	Adipose, hepatic, and systemic inflammation	[31]
Nrf2↑→insulin resistance↓	Nrf2 agonist: resveratrol	Hepatic oxidative stress	[33]
Nrf2↑→insulin resistance↓	Nrf2 agonist: resveratrol	Extracellular signal regulated kinase (ERK) pathway	[47]
Nrf2↑→insulin resistance↓	Nrf2 agonist (Dh404)	ERK pathway	[48]
